# Meropenem‐Heparin Lock Therapy for *Ochrobactrum intermedium* Catheter‐Related Bloodstream Infection in a Pediatric Hemodialysis Patient: A Case Report and Literature Review

**DOI:** 10.1111/hdi.70050

**Published:** 2026-01-06

**Authors:** Mônica Cristina Dutra Rodrigues, Fernanda Teixeira Sillva, Anderson Pereira de Oliveira, Yeo Jim Kinoshita Moon, Jhohann Richard de Lima Benzi

**Affiliations:** ^1^ Faculty of Pharmaceutical Sciences of Ribeirão Preto University of Sao Paulo Ribeirão Preto São Paulo Brazil; ^2^ Federal University of Alfenas Alfenas Minas Gerais Brazil; ^3^ Federal University of Rio Grande Do Norte, Campus Universitário‐Lagoa Nova Natal Rio Grande do Norte Brazil; ^4^ Menino Jesus Municipal Children's Hospital São Paulo São Paulo Brazil; ^5^ School of Pharmaceutical Sciences University of São Paulo São Paulo São Paulo Brazil

**Keywords:** antimicrobial lock therapy, catheter‐related infection, hemodialysis, heparin, *Ochrobactrum*, pediatric

## Abstract

**Background:**

In pediatric hemodialysis patients with exhausted vascular access, managing rare pathogens like *Ochrobactrum intermedium* is challenging, often posing a dilemma between catheter removal and salvage attempts. There are few reports on the supported viability of meropenem‐heparin lock therapy for this purpose in pediatric patients.

**Case Presentation:**

We present the case of a 12‐year‐old male with chronic kidney disease on hemodialysis who was admitted with sepsis attributed to her long‐term hemodialysis catheter. *Ochrobactrum intermedium* was isolated from all catheter lumens and blood cultures. Due to a documented history of vascular access failure, catheter salvage was the primary goal. A combined systemic antibiotic and lock therapy with meropenem and unfractionated heparin was initiated. Critically, bacteremia relapsed promptly after a planned temporary suspension of the lock therapy, while systemic antibiotics were continued. Definitive microbiological eradication was only achieved after the re‐initiation and completion of a 14‐day course of the lock therapy, resulting in full clinical recovery and long‐term catheter preservation. This case report suggests the potential efficacy of meropenem lock therapy as a salvage strategy for *O. intermedium* CRBSI when catheter removal is not a viable option. The relapse upon its suspension highlights that the lock therapy itself was the critical component for eradicating the *O. intermedium* biofilm, a feat the systemic antibiotic alone could not achieve. This approach is a viable alternative when catheter removal is not feasible.

## 1 Introduction

Catheter‐related bloodstream infections (CRBSI) represent a major cause of morbidity and hospitalization in the pediatric dialysis population, significantly impacting patient survival and quality of life [1, 2]. While guidelines prioritize catheter removal for specific pathogens, this approach is complicated in children with exhausted vascular access [3].

The difficulty in treating CRBSI lies in the formation of biofilm on catheter surfaces, which significantly hinders antibiotic penetration, rendering systemic therapy alone often insufficient for eradication. In this context, antimicrobial lock therapy has emerged as a crucial adjuvant strategy [4]. This therapy aims to eradicate the biofilm and sterilize the catheter, facilitating device salvage when combined with systemic therapy [5].

Although most CRBSI are caused by Gram‐positive cocci, infections by Gram‐negative bacilli (GNB), such as those of the *Ochrobactrum* genus, are increasing in frequency and represent a significant therapeutic challenge. *Ochrobactrum* spp. are emerging opportunistic pathogens known for their environmental ubiquity, capacity to form biofilm, and a profile of intrinsic resistance to multiple antibiotic classes, including many cephalosporins and penicillins [6].

There is a lack in the literature about the management of CRBSI caused by *Ochrobactrum intermedium* , specifically in pediatric hemodialysis patients. Furthermore, there is an absence of data validating the use of specific lock therapies for this rare pathogen. Therefore, we present a case report of the successful management of *O. intermedium* CRBSI in a pediatric patient using a meropenem‐heparin lock therapy, and we conduct a literature review on the pathogen and its treatment strategies. To our knowledge, this is the first report describing the use of antimicrobial lock therapy for *O. intermedium* in a pediatric hemodialysis patient.

## 2 Case Report

We present a case of a 12‐year‐old male patient (26.6 kg) with nephrotic syndrome and chronic kidney disease, status‐post kidney transplantation in 2020, which resulted in graft rejection. He has been on hemodialysis three times a week for the past 5 years, currently via a Permcath catheter inserted in the right internal jugular vein on August 23, 2024. In addition to clinical fragility, the patient has a history of difficulties with vascular access, which limits the possibility of safely changing the catheter and imposes restrictions on invasive management. Although catheter removal is the gold‐standard therapy for CRBSI, this approach was clinically contraindicated. The patient had a documented history of exhausted vascular access, and peritoneal dialysis (PD) was also contraindicated due to significant psychosocial barriers, making in‐center hemodialysis the only viable renal replacement therapy (RRT) modality. The social and family support is limited, the grandmother being his primary caregiver. The patient was admitted on February 19 due to fever and chills associated with the use of the hemodialysis catheter during the last session performed on the same day. Due to the reduced level of consciousness associated with sepsis and worsening of renal parameters, as well as the need for hemodynamic monitoring, the patient was intubated and transferred to the Intensive Care Unit (ICU). On the same day, upon admission, blood cultures were collected from both sides of the Permcath catheter (MedCorp) and peripherals, and the Permcath catheter was isolated for non‐use. Antibiotic therapy was initiated with meropenem at 20 mg/kg once daily intravenously, adjusted for renal function, and vancomycin at 10 mg/kg on dialysis days, adjusted based on therapeutic drug monitoring using the area under the curve (AUC) method, targeting an AUC/MIC of 400–600. Laboratory tests showed a normal leukocyte count (5000–17,000/mm^3^) on admission; however, C‐reactive protein was elevated at 16.40 mg/L. Significant renal and hepatic abnormalities were observed, including aspartate aminotransferase (AST): 185 U/L, alanine aminotransferase (ALT): 81 U/L, gamma‐glutamyl transferase (GGT): 152 U/L, serum creatinine: 10.40 mg/dL, and urea: 173 mg/dL. On the second day of hospitalization, the patient was extubated and continued to have vital signs monitored and undergo hemodialysis according to daily needs. On the fifth day of hospitalization, the patient had a fever, which repeated more frequently in the following days until the beginning of March, and blood cultures were then collected from the central and peripheral arteries. On March 4, a gram‐negative bacillus (GNB) growth was observed in the catheter blood culture (collected on February 28), and systemic treatment was initiated, with ciprofloxacin 15 mg/kg/day IV in the Permcath lock therapy. The following day, the patient remained stable, although the catheter culture showed growth of GNB in both lumens of the Permcath.

Based on the patient's evolving condition and initial microbiological data, an empirical switch was made to ciprofloxacin (15 mg/kg/day IV) on March 4th, which was also instituted as the lock therapy. Despite this change, the patient's condition deteriorated, with persistent bacteremia observed by the end of the day.

After consultation with the infectious disease team on March 6, systemic therapy was changed to levofloxacin IV (10 mg/kg every 48 h, adjusted for hemodialysis).

Subsequently, the final antimicrobial susceptibility testing results were reported for the *Ochrobactrum intermedium* isolate (from the blood culture collected on February 28 from both Permcath routes). The susceptibility testing results revealed a challenging, limited‐sensitivity profile: the isolate was susceptible only to meropenem, imipenem, and levofloxacin with minimum inhibitory concentration (MIC) 0.38 mg/L, 1.0 and 0.125 mg/L, consecutively. See Table 1 for the chronological sequence of cultures and sensitivities.

**TABLE 1:** Chronological summary of the evolution of the clinical case.

Date	Clinical/Laboratory/Therapeutic event
February 19	Patient hospitalization due to fever and chills following hemodialysis. Blood cultures were obtained from both the catheter and peripheral sites. The Permcath catheter was isolated and discontinued from use. The patient was intubated and transferred to the ICU. Empirical antimicrobial therapy was initiated with meropenem (20 mg/kg/day IV) and vancomycin (10 mg/kg IV on dialysis days). Laboratory tests revealed impaired renal and hepatic function, along with elevated CRP levels.
February 20	Extubation performed. The patient remains on hemodialysis and under continuous monitoring.
~Around February 24 (day 5)	Febrile peak observed. Repeat blood cultures were collected from both central and peripheral sites. Fever episodes became more frequent.
March 4	Blood culture result from February 28: growth of GNB in the catheter sample. Intravenous ciprofloxacin was initiated (15 mg/kg/day), along with ciprofloxacin lock therapy in the Permcath.
March 5	Catheter culture positive for GNB in both lumens. Intravenous ciprofloxacin and lock therapy were maintained. Bacteremia was detected by the end of the day.
March 6	Ciprofloxacin was switched to intravenous levofloxacin (10 mg/kg every 48 h). Blood culture results from February 28 identified *Ochrobactrum intermedium* , susceptible to meropenem, imipenem, and levofloxacin. The clinical pharmacy team contraindicated levofloxacin lock therapy due to the risk of physicochemical incompatibility.
March 7	The decision was made to maintain the catheter and initiate lock therapy with meropenem plus unfractionated heparin in both lumens of the Permcath. Daily preparation and handling were performed according to the instructional protocol developed by the clinical pharmacy team. Pharmacy, nursing, and ICU staff were trained accordingly.
After March 7	Lock therapy with meropenem plus heparin was maintained daily until clinical resolution. Continuous clinical monitoring was performed.

Abbreviations: CRP, C‐reactive protein; GNB, gram‐negative bacillus; ICU, intensive care unit; IV, intravenous.

This critical result report confirmed the inadequacy of the interim ciprofloxacin therapy and validated the switch to systemic levofloxacin. However, it created a challenge for the lock therapy. The clinical pharmacy team advised against using a levofloxacin lock, citing a scarcity of supporting literature and a high risk of physicochemical incompatibility, which could lead to catheter loss.

Given the patient's continued fever, clinical instability, and the difficulty of obtaining new vascular access, a multidisciplinary decision was made on March 7 to salvage the catheter. The agreed‐upon strategy was to continue systemic levofloxacin (as guided by the susceptibility testing results) and initiate a different antibiotic lock. Meropenem was selected for the lock component, based on literature reports supporting its stability and efficacy in lock solutions.

Following this interdisciplinary decision, the hospital pharmacy began compounding the meropenem‐heparin lock solution. The preparation followed a standardized protocol: one 500 mg vial of meropenem was reconstituted with 10 mL of sterile water for injection (50 mg/mL). A 1 mL aliquot (50 mg) was further diluted in 24 mL of 0.9% sodium chloride to an intermediate concentration of 2 mg/mL. Finally, 0.4 mL of unfractionated heparin (5000 IU/mL), corresponding to 2000 IU, was added to 1 mL of the intermediate meropenem solution.

The final mixture was drawn into two sterile 2 mL syringes, one for each lumen of the Permcath. This preparation was sent daily to the ICU. The solution was designated for same‐day use, with a maximum 24‐h post‐compounding stability, and was replaced daily to ensure efficacy.

Systemic treatment with intravenous levofloxacin was maintained concurrently for 7 days, and the patient continued to undergo hemodialysis with daily blood culture monitoring from both catheter lumens.

Table 1 presents the timeline of these critical events, laboratory results, and therapeutic interventions, providing a systematized view of the case evolution.

## 3 Clinical Evolution and Results

The response to the antibiotic lock therapy was monitored using clinical and laboratory parameters. A significant clinical improvement was observed, with resolution of fever and chills. After 7 days of lock therapy (by March 14), the C‐reactive protein level decreased significantly from 16.40 to 1.20 mg/dL.

Control blood cultures drawn prior to this date had resulted in partial negative, and the catheter remained functional without signs of local infection or permeability issues. Given this marked improvement, the meropenem‐heparin lock therapy was discontinued on March 14.

## 4 Clinical Relapse and Re‐Challenge

Despite clinical improvement, a prompt microbiological relapse followed. New blood cultures drawn on March 17 (3 days after antimicrobial lock therapy discontinuation) were again positive for GNB growth. An echocardiogram performed at the time of this relapse was normal, ruling out new‐onset endocarditis. This sequence strongly indicated that while systemic therapy was insufficient, the lock therapy was essential for controlling the biofilm.

On March 18, *Ochrobactrum intermedium* was isolated again in both Permcath lines. Therefore, on the same day, it was advised to resume lock therapy with meropenem + unfractionated heparin, with daily exchange and following the previous guidance; however, it was advised to maintain lock therapy for at least 14 days. It was also advised to collect control blood cultures at least every 48‐72 h.

## 5 Clinical and Laboratory Evidence of Response to Treatment With Lock Therapy

Treatment evolution was monitored by clinical and laboratory parameters. Associated with the cessation of fever and chills, the maintenance of infectious parameters within normal laboratory tests (C‐reactive protein and leukocytes) was observed, and the control blood cultures collected on March 22, 24, and 27 showed the eradication of bacterial growth of *Ochrobactrum intermedium* . No adverse events related to lock therapy or systemic treatment were observed.

The catheter remained patent with evidence of systemic and local treatment of permcath by combined lock therapy of meropenem with unfractionated heparin. After 14 days of reintroduction of treatment with combined lock therapy of meropenem + unfractionated heparin, on March 31, the patient was discharged from hospital with the permcath functioning and all control blood cultures negative.

Below, the tables illustrate the clinical evolution of the case. While Table 1 summarizes the chronology of key clinical interventions, Table 2 focuses on the laboratory data that substantiates this evolution. The data is organized to demonstrate the direct correlation between the therapy and the patient's inflammatory response, illustrating the case's three distinct phases: the initial improvement with lock therapy, the immediate relapse upon its discontinuation, and the definitive resolution after its reinitiation.

**TABLE 2:** Evolution of key laboratory parameters during hospitalization.

Parameter (unit)	Feb 19 (Admission)	Feb 28 (1st isolated)	Mar 7 (Lock Start)	Mar 12 (2nd isolated)	Mar 14 (Lock Stop)	Mar 17 (Relapse)	Mar 18 (3rd isolated)	Mar 28 (Eradication)	Mar 31 (Discharge)	Reference Range
Inflammatory markers										
CRP (mg/dL)	16.4	2	2.4	0.8	1.2	0.5	0.5	0.5	0.7	< 1
Leukocytes (×10^3^/μL)	5.5	5.0	3.9	3.6	3.2	4.4	3.8	4.2	3.4	4.5–14.5
Neutrophils (%)	59	63	42.3	45	37.3	39.4	31	29.3	38	40–60
Lactato (mmol/L)	—	—	0.7	1.0	2.5	0.9	1.7	1.3	1.1	0.5 a 1.6
Hematology										
Hemoglobin (g/dL)	9.6	9.3	8.5	7.3	9.5	10.2	9.9	10.7	10.3	11.5–15.5
Platelets (×10^3^/μL)	135	153	230	162	161	170	167	153	139	150–400
Biochemistry										
Creatinine (mg/dL)	10.4	9.7	7	8.7	9.7	9.9	8.1	—	9.4	0.4–1.0
AST (U/L)	185	37	—		—	—	—	—	—	15–40
ALT (U/L)	81	10	—	—	—	—	—	—	—	< 50
Microbiology										
Blood culture (Central)	Neg.	*O.i*.	O.i.	*O.i*.	GNB	GNB	*O.i*.	Neg.	Neg.	Neg.
Blood culture (Periph.)	Neg.	Neg.	Neg.	Neg.	Neg.	Neg.	Neg.	Neg.	Neg.	Neg.

Abbreviations: ALT, alanine aminotransferase; AST, aspartate aminotransferase; CRP, C‐reactive protein; GNB, gram‐negative bacillus; Neg, negative; *O.i*., *Ochrobactrum intermedium* ; Pos, positive.

## 6 Discussion

This clinical course supports the hypothesis that antimicrobial lock therapy may be a viable alternative when catheter removal is not an option. Lock therapy, combining antibiotics and anticoagulants, has been explored as a strategy for in situ infection eradication and catheter preservation, with promising outcomes reported in specific clinical scenarios [1].

The success of this management, however, is non‐trivial and lies at the intersection of three clinical challenges, which this discussion will now address: the pathogen, the intervention, and the patient scenario.

### 6.1 The Pathogen: *Ochrobactrum intermedium*

The first challenge is the pathogen itself. This case report highlights the identification of *Ochrobactrum intermedium* , a rare, non‐fermenting GNB, notable for its emergence in immunocompromised patients and the associated therapeutic difficulty [6]. Recent studies indicate that *Ochrobactrum intermedium* exhibits intrinsic resistance to diverse antibiotic classes, with carbapenems, such as meropenem, being the most effective agents against this pathogen [6, 7].

The literature corroborates this challenge; a comprehensive review by Ryan and Pembroke on the *Ochrobactrum* genus identified its clear association with medical devices and hospital environments, its multidrug‐resistance profile, and its capacity to form biofilm [6, 8]. Its notorious capacity to form persistent biofilm on plastic surfaces, such as in catheter lumens [9], underscores the necessity of an intensive local approach, given that systemic therapy alone is frequently ineffective in eradicating the biofilm.

Reports of infections caused by *O. intermedium* are rare and generally associated with bacteremia in immunocompromised patients [6, 10, 11], whereas *Ochrobactrum anthropi* is more frequently found as the infectious agent [12]. Literature on infections caused by *O. intermedium* is scarce. To date, only 11 case reports of *O. intermedium* ‐related infections have been described [10, 11, 13, 14, 15, 16, 17, 18, 19, 20, 21], with only one of these occurring in a pediatric patient [10]. In general, infections by *Ochrobactrum* spp. are typically associated with immunocompromised patients, presenting as bacteremia, endophthalmitis, and pyogenic liver abscess [13, 14, 15, 16, 17].

### 6.2 Antimicrobial Lock Therapy as a Salvage Strategy

This introduces the second challenge: biofilm eradication. The failure of systemic therapy alone in eradicating CRBSI is directly linked to biofilm formation, which demands antibiotic concentrations 100–1000 times higher than the MIC. Antimicrobial lock therapy is designed to overcome this barrier. Recent systematic and scoping reviews by Aksoy et al. [4], Alfieri et al. [5], and Tresso et al. [22] confirm that antimicrobial lock therapy, as an adjuvant to systemic therapy, demonstrates high catheter‐salvage success rates (frequently > 70%) and is considered an effective strategy in clinical practice when catheter removal is not viable.

### 6.3 Antimicrobial Lock Therapy in Pediatrics and the Stability of Meropenem‐Heparin

The third challenge is the application of this therapy in the pediatric setting. In the pediatric hemodialysis population, antimicrobial lock therapy is a “highly attractive adjunct treatment option” due to the critical need to preserve vascular access [1]. However, as noted by Báez‐Gutiérrez et al. [23], the Infectious Diseases Society of America (IDSA) guidelines do not include specific measures for pediatric patients [11]. This makes antimicrobial lock therapy a challenge that demands careful evaluation of stability, compatibility, and the risk of systemic toxicity.

The use of meropenem in pediatric/neonatal antimicrobial lock therapy is cited in the literature; Piersigilli et al. reported its successful use in neonates, confirming its efficacy and stability for the lock [3]. The choice of meropenem in our case was based on its good biofilm penetration and its solution stability profile for up to 24 h [22, 23].

The addition of heparin, despite its traditional use to maintain catheter patency, remains poorly documented in association with antibiotics in pediatrics [22, 23, 24, 25]. The literature, although scarce, suggests the combination can be safe and effective, provided it is handled with rigorous aseptic technique and respects physicochemical stability limits. In our case, the daily preparation of the solution, based on hospital guidelines and protocols, ensured the microbiological safety and stability of the compound.

### 6.4 The Convergence of Clinical Challenges

These three challenges (a rare, biofilm‐forming pathogen; the necessity of antimicrobial lock therapy for biofilm; and the complexity of pediatric antimicrobial lock therapy) converged in our patient. The decision for catheter salvage, rather than removal, was clinically mandatory due to the patient's exhausted vascular access. The patient had a documented history of multiple thrombotic events at previous access sites, as well as repeated puncture failures at contralateral jugular and femoral sites. Consequently, any attempt to place a new central access was deemed prohibitively high‐risk, establishing the clear clinical imperative to preserve the existing catheter.

The PD, while an established RRT modality for pediatrics, was formally contraindicated in this case due to psychosocial barriers and low health literacy. PD is a home‐based therapy that demands rigorous caregiver training for aseptic exchanges, supply management, and the early identification of complications, such as peritonitis.

In this patient's scenario, a home support structure with significant limitations was identified. The primary caregiver presented with low health literacy, with documented difficulties in adhering to and managing the patient's complex oral polypharmacy regimen. It was assessed that the technical complexity and rigorous aseptic demands of PD posed a prohibitive risk of technical failure and severe infectious complications. Therefore, in‐center hemodialysis was maintained as the only viable RRT modality, reinforcing the critical need to preserve the existing vascular access.

This case, therefore, should not be viewed as a deviation from the documented and clinical standard, but rather as the successful implementation of an essential salvage therapy. The approach was dictated by a complex clinical scenario where options were severely limited.

The most robust evidence supporting the efficacy of the lock therapy in this case comes from the clinical course observed after its planned suspension. The lock therapy was interrupted on March 14, which was promptly followed by a clear microbiological relapse: *Ochrobactrum intermedium* was re‐isolated from both Permcath lumens on March 17 and 18. Definitive microbiological eradication, confirmed by multiple subsequent negative blood cultures, was only achieved after the re‐initiation and completion of the lock therapy for a full 14‐day course. This sequence strongly suggests that while systemic antibiotics were a necessary adjuvant, the lock therapy itself was the essential component for eradicating the catheter biofilm—a feat that systemic therapy alone failed to achieve.

### 6.5 Identified Gap

When comparing our findings with the literature, a recent meta‐analysis of pediatric data [26] and a single‐center study [27] have shown that the addition of antimicrobial lock therapy is superior to systemic antibiotics alone for treating CRBSI and was also associated with lower recurrence, particularly when linked to rigorous infection control practices and laboratory monitoring. However, the majority of published data either focuses on adults or does not address rare Gram‐negative pathogens [28, 29, 30, 31, 32, 33, 34, 35, 36, 37, 38, 39, 40, 41, 42, 43, 44, 45, 46, 47, 48, 49, 50, 51, 52, 53, 54, 55, 56].

To specifically contextualize the novelty of our case, we conducted a literature review seeking published cases of infections where *Ochrobactrum* spp. was isolated in pediatric patients, as well as their treatment method (Table 3). Our review identified no published cases of *O. intermedium* CRBSI treated with meropenem‐heparin antimicrobial lock therapy in a pediatric hemodialysis patient. This positions our report as a significant contribution, providing unprecedented data on the management of a rare infection in a high‐complexity clinical scenario.

**TABLE 3:** Review of published cases of *Ochrobactrum* spp. infections in pediatric patients.

Author (Year)	Ochrobactrum species	Sex/Age	Underlying condition	Type of infection	Use of lock therapy?	Outcome
Barson et al. (1987) [30]	*O. anthropi*	M/14 years	Puncture wound (foot)	Osteochondritis	None (Systemic only)	Complete recovery
Cieslak et al. (1992) [31]	*O. anthropi*	F/3 years	Retinoblastoma	CRBSI	None (Systemic only)	Complete recovery
Klein & Eppes (1993) [32]	*O. anthropi*	F/7 years	Leukemia	CRBSI	Gentamicin, Imipenem + Catheter removal	Complete recovery
Haditsch et al. (1994) [33]	*O. anthropi*	F/14 years	Leukemia	Bacteremia	N/A	Complete recovery
Jelveh & Cunha (1999) [34]	*O. anthropi*	M/33 months	Osteomyelitis	Bacteraemia	N/A	Complete recovery
Hay & Lo (1999) [35]	*O. anthropi*	F/Neonate	Neonate	Meningitis	None (Systemic only)	Complete recovery
Saavedra et al. (1999) [36]	*O. anthropi*	M/4 years old	Neuroblastoma	CRBSI	None (Systemic only)	Complete recovery
Blecker & Mortensen (2000) [37]	*O. anthropi*	M/2.5 years old	None	Infection	None (Systemic only)	Complete recovery
Galanakis et al. (2002) [38]	*O. anthropi*	(11 Cases—All < 7 years old)	None	Bacteraemia	None (Systemic only)	Complete recovery
Stiakaki et al. (2002) [39]	*O. anthropi*	(11 Cases—All < 11 years old)	None	CRBSI	Systemic antibiotics + Catheter removal (4 cases)	Complete recovery
Gascón et al. (2003) [40]	*O. anthropi*	M/3 years old	None	Bacteraemia	None (Systemic only)	Complete recovery
Aly et al. (2007) [41]	*O. anthropi*	F/2 years old	LCHAD deficiency	Bacteraemia	None (Systemic only)	Complete recovery
Battaglia et al. (2008) [42]	*O. anthropi*	M/17 years old	None	Septic arthritis	None (Systemic only)	Complete recovery
Menuet et al. (2008) [43]	*O. anthropi*	F/17 years old	Cystic Fibrosis, Diabetes	Pneumonia	None (Systemic only)	Complete recovery
Duran et al. (2009) [44]	*O. anthropi*	M/Neonate	Neonate (meconium peritonitis)	CRBSI	None (Systemic only)	Died (Unrelated to *Ochrobactrum* infection)
Yagüe‐Muñoz et al. (2010) [45]	*O. anthropi*	M/8 years old	Cystic fibrosis	Bacteraemia	None (Systemic only)	Complete recovery
Obando et al. (2011) [46]	*O. anthropi*	F/19 years old	Hypothyroidism, ESRD	CRBSI	Levofloxacin + Catheter removal	Complete recovery
Alparslan et al. (2013) [47]	*O. anthropi*	M/12 years old	ESRD	Peritonitis + PD	Systemic antibiotics + Catheter removal	Complete recovery
Mudshingkar et al. (2013) [48]	*O. anthropi*	M/Neonate	Neonate	Septicaemia	None (Systemic only)	Died
Al‐Naami et al. (2014) [49]	*O. anthropi*	M/15 years old	None	Retropharyngeal abscess	N/A	Complete recovery
Hernández‐Torres et al. (2014) [50]	*O. anthropi*	5 Months old	None	Pseudo‐bacteraemia	N/A	N/A
Menezes et al. (2014) [51]	*O. anthropi*	F/Neonate	Neonate with Cystic Fibrosis	CRBSI	None (Systemic only)	Complete recovery
Qasimyar et al. (2014) [52]	*O. anthropi*	M/Neonate	Neonate	Sepsis (catheter‐related)	None (Systemic only)	Complete recovery
Cenkçi et al. (2015) [53]	*O. anthropi*	F/13 months old	None	Bacteremia, pneumonia	None (Systemic only)	Complete recovery
Gigi et al. (2017) [54]	*O. anthropi*	M/18 years old	None	Osteomyelitis (Foot puncture)	None (Systemic only)	Complete recovery
Khasawneh & Yusef (2017) [55]	*O. anthropi*	F/Neonate	Neonate	Sepsis (catheter‐related)	None (Systemic only)	Complete recovery
Grabowska‐Markowska et al. (2019) [56]	*O. anthropi*	M/13 years old	Neurodegenerative disorder	Bacteraemia	None	Complete recovery
Wu et al. (2021) [10]	*O. intermedium*	M/2 years	Pineoblastoma	Bacteremia	Systemic antibiotics + Catheter removal	Complete recovery
Our Case (2025)	*O. intermedium*	M/12 years	ESRD (nephropathic syndrome) on Hemodialysis	CRBSI	Systemic antibiotics + Lock therapy (meropenem + heparin)	Complete recovery and catheter salvage

Abbreviations: CRBSI, Catheter‐related bloodstream infection; ESRD, end‐stage renal disease; LCHAD, long‐chain 3‐hydroxyacyl‐coenzyme A dehydrogenase deficiency; PD, peritoneal dialysis.

To our knowledge, this is the first clinical case report of *O. intermedium* bloodstream infection in a pediatric hemodialysis patient.

### 6.6 Barriers and Prognosis

During the management of this case, several clinical and therapeutic difficulties emerged. The primary challenge was the necessity of maintaining the hemodialysis catheter (Permcath) despite the associated recurrent bacteremia, given the absence of viable alternative vascular access. Another barrier was the scarcity of literature data regarding the efficacy and safety of lock therapy for this specific pathogen, requiring clinical decisions to be based on interdisciplinary consensus.

This case, therefore, reinforces the importance of collaborative action among the infectiology, nephrology, clinical pharmacy, and nursing teams to individualize treatment, safely manage the lock therapy, and monitor the clinical response. It also highlights the relevance of pharmacovigilance and the stability of compounded solutions, particularly in the pediatric hospital setting. Regarding prognosis, despite clinical stabilization and discharge following therapeutic adjustments, the patient remains at high risk for CRBSI recurrence.

This is especially true given the underlying condition (nephrotic syndrome with immunosuppression and chronic renal failure) and the chronic dependence on hemodialysis via central access. This makes continuous monitoring and microbiological surveillance essential.

Finally, the decision to proceed with salvage therapy was perceived by the patient's legal guardian as a significant gain in quality of life, as it avoided more invasive and painful procedures. The patient continues to be monitored in ambulatory follow‐up.

It is important to acknowledge the limitations of this report. As a single‐case observation, the findings may not be generalizable to the broader pediatric population. Additionally, the lack of standardized protocols for *O. intermedium* treatment and the specific concentration of meropenem for lock therapy warrant caution. Further studies are needed to establish optimal dosing and duration.

## 7 Conclusion

In conclusion, antimicrobial lock therapy represents a critical salvage intervention for pediatric patients when conventional management involving catheter removal is precluded by the exhaustion of vascular access sites. This report highlights the clinical significance of *Ochrobactrum intermedium* as an emerging, multi‐resistant pathogen in the dialysis setting, emphasizing that successful eradication may require a combined biofilm‐targeting approach. While this case provides a valuable therapeutic template, the lack of standardized pediatric protocols necessitates further prospective data and larger‐scale studies to validate these findings in the broader pediatric hemodialysis population.

Informed consent for publication of this case report was obtained from the patient's legal guardian, as well as approval from a specific ethics committee (CAAE 89408825.9.0000.5639).

## Disclosure

The authors have nothing to report.

## Conflicts of Interest

The authors declare no conflicts of interest.

## Data Availability

Data sharing not applicable to this article as no datasets were generated or analysed during the current study.
